# Heterologous SUMO-2/3-Ubiquitin Chains Optimize IκBα Degradation and NF-κB Activity

**DOI:** 10.1371/journal.pone.0051672

**Published:** 2012-12-20

**Authors:** Fabienne Aillet, Fernando Lopitz-Otsoa, Isabel Egaña, Roland Hjerpe, Paul Fraser, Ron T. Hay, Manuel S. Rodriguez, Valérie Lang

**Affiliations:** 1 Proteomics Unit, CIC bioGUNE, CIBERehd, Derio, Bizkaia, Spain; 2 Ubiquitylation & Cancer Molecular Biology Laboratory, Inbiomed, San Sebastián-Donostia, Gipuzkoa, Spain; 3 Tanz Centre for Research in Neurodegenerative Diseases and Department of Medical Biophysics, University of Toronto, Ontario, Canada; 4 Centre for Interdisciplinary Research, School of Life Sciences, University of Dundee, Dundee, Scotland, United Kingdom; University of Hong Kong, Hong Kong

## Abstract

The NF-κB pathway is regulated by SUMOylation at least at three levels: the inhibitory molecule IκBα, the IKK subunit γ/NEMO and the p52 precursor p100. Here we investigate the role of SUMO-2/3 in the degradation of IκBα and activation of NF-κB mediated by TNFα. We found that under conditions of deficient SUMOylation, an important delay in both TNFα-mediated proteolysis of IκBα and NF-κB dependent transcription occurs. *In vitro* and *ex vivo* approaches, including the use of ubiquitin-traps (TUBEs), revealed the formation of chains on IκBα containing SUMO-2/3 and ubiquitin after TNFα stimulation. The integration of SUMO-2/3 appears to promote the formation of ubiquitin chains on IκBα after activation of the TNFα signalling pathway. Furthermore, heterologous chains of SUMO-2/3 and ubiquitin promote a more efficient degradation of IκBα by the 26S proteasome *in vitro* compared to chains of either SUMO-2/3 or ubiquitin alone. Consistently, Ubc9 silencing reduced the capture of IκBα modified with SUMO-ubiquitin hybrid chains that display a defective proteasome-mediated degradation. Thus, hybrid SUMO-2/3-ubiquitin chains increase the susceptibility of modified IκBα to the action of 26S proteasome, contributing to the optimal control of NF-κB activity after TNFα-stimulation.

## Introduction

Protein modification with ubiquitin and the ubiquitin-like protein SUMO regulates a large diversity of cellular processes including cell cycle, apoptosis, DNA repair and signal transduction pathways [1]. The attachment of ubiquitin to a substrate, commonly known as ubiquitylation, involves the action of at least three enzymes, a ubiquitin activating enzyme or E1, a conjugating enzyme or E2 and a ubiquitin ligase or E3 [2]. The attachment of one of the three SUMO modifiers (SUMO-1, SUMO-2, SUMO-3) to a target protein (SUMOylation), is a biochemical process similar to ubiquitylation but involving SUMO specific E1, E2 and E3 enzymes [1]. Ubiquitin can be attached as a monomer in a single (monoubiquitylation) or multiple moieties (multiple monoubiquitylation). Ubiquitin can also form polymers of complex composition through the attachment of additional ubiquitin molecules on any of the seven lysine-residues present in each ubiquitin. Canonical functions have been attributed to some of these chain types. Chains linked through lysine 48 (K48) and 11 (K11) are mainly associated to protein degradation [2] meanwhile K63 and linear chains are associated to signal transduction [3], [4], [5], [6], [7], [8]. However, chain composition appears to be more complex since mixed chains [9], [10] as well as heterologous chains including other ubiquitin-like molecules such as SUMO-2/3 have been found [11], [12]. Ubiquitylation and SUMOylation are highly dynamic reversible processes where deconjugation is mediated by a set of enzymes generically named deubiquitylating enzymes (DUBs) or SUMO-specific proteases (SUSPs) respectively [13], [14], [15].

The NF-κB pathway, one of the best-characterized signalling pathways regulated by ubiquitylation [16], leads to a variety of cellular responses, including the induction of pro-inflammatory and anti-apoptotic genes. One of the most abundant forms of NF-κB in mammals is a heterodimer composed of p65 and p50, whose activity is tightly controlled by a family of natural inhibitors named IκBs (α, β and ε) [17]. In addition to ubiquitylation, this pathway is controlled by many other post-translational modifications including SUMOylation, NEDDylation, phosphorylation, and acetylation. These frequently have distinct, sometimes antagonistic, functional consequences [18], [19], [20], [21]. Regulation by these posttranslational modifications can occur at different levels of the signalling cascade controlling NF-κB activation, including the activation of the essential IκB kinase IKK [22], maturation of the p50 precursor p105 [23], modification of NF-κB subunits and IκB molecules [16]. IκBα is modified with SUMO-1, which competes with ubiquitin for the same acceptor lysine (K21) during signal-mediated stimulation [21]. While polyubiquitylation of IκBα depends on the IKK-mediated phosphorylation of serines 32 and 36 for its subsequent recognition by the ubiquitin-ligase (E3) SCF-βTrCP, IκBα SUMOylation with SUMO-1 does not depend on its phosphorylation [21]. A SUMO E3 ligase for IκBα has not been reported, but the unique E1 (SAE) and E2 (Ubc9) are sufficient for its SUMOylation *in vitro* [21]. As for other target proteins [13], [15], IκBα ubiquitylation and SUMOylation are tightly controlled by the action of unidentified DUBs and SUSPs. In addition to IκBα, other proteins involved in the NF-κB signalling pathway are modified with SUMO (IKKγ/NEMO, IKKε and p100) [22], [24]. Recently, it has been demonstrated that SUMOylation with SUMO-2 can promote ubiquitylation by ubiquitin E3s and therefore simultaneous substrate modification with both ubiquitin and SUMO proteins is possible [11], [12]. Using *in vitro* and *ex vivo* approaches, we investigate the role of SUMO-2 and SUMO-3 in the TNFα-induced IκBα degradation and the activation of the NF-κB transcription factor. We found that SUMO-2/3 forms heterologous chains with ubiquitin on IκBα, contributing to its optimal proteasomal degradation. This reveals an unsuspected importance of hybrid chains in TNFα mediated proteolysis of IκBα and subsequent activation of NF-κB promoted transcription.

## Results

### SUMOylation Contributes to the Optimal TNFα-mediated NF-κB Activation and Degradation of IκBα

**Figure 1:**
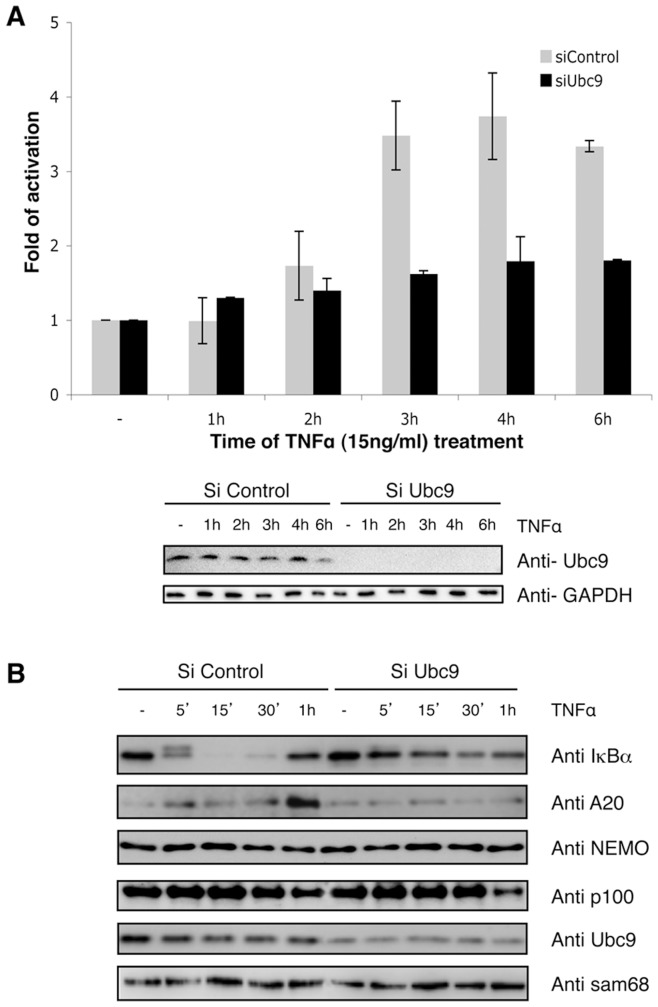
SUMOylation contributes to the optimal TNFα-mediated NF-κB activation and degradation of IκBα. (A) HeLa cells were transfected 72 h with control or Ubc9 siRNA (100 nM). Cells were co-transfected with a NF-κB-luciferase reporter plasmid (3EnhancerConA) and β-galactosidase reporter. Twenty-four hours later cells were stimulated with TNFα (15 ng/ml) as indicated and luciferase and β-galactosidase activities measured as previously described [50]. The graph corresponds to the mean of three independent experiments. (B) HeLa cells were transfected during 72 h with control or Ubc9 siRNA (100 nM) and stimulated with TNFα (15 ng/ml) as indicated. Western-blot analyses were performed with the indicated antibodies.

To evaluate the contribution of SUMOylation in TNFα-induced activation of NF-κB, Ubc9 silencing experiments were performed in HeLa cells (Figure 1). A clear defect of NF-κB activation was observed using an NF-κB luciferase-reporter assay (Figure 1A) or expression of NF-κB-dependent proteins such as A20 and IκBα (Figure 1B). More importantly, we also observed a clear defect in TNFα-mediated phosphorylation and degradation of IκBα at the early stage of stimulation (around 5–15 minutes) just prior to the maximal degradation [25], [26] (Figure 1B). To investigate if other known SUMO substrates of the NF-κB were affected at the level of protein stability, western blots against p100 and NEMO/IKKγ were performed (Figure 1B). Our results indicate that IκBα is the only analysed SUMO target of the NF-κB pathway affected at the level of protein stability. Thus, attenuated IκBα SUMOylation correlates with a deficient TNFα-induced IκBα degradation and NF-κB activation.

### IκBα is Modified by SUMO-2/3 *in vitro* and *ex vivo*

**Figure 2:**
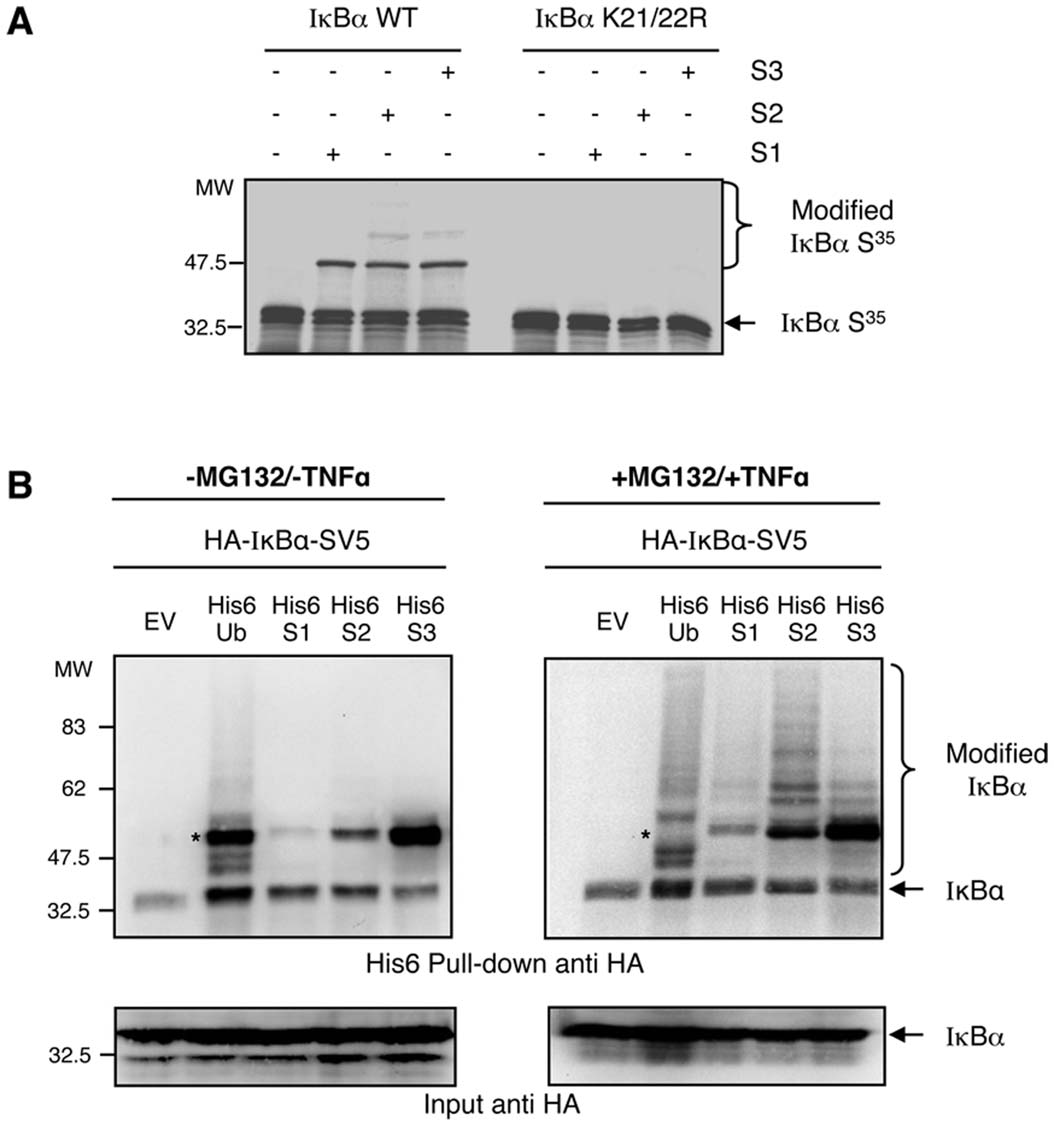
IκBα is modified by SUMO-2/3 *in vitro* and *ex vivo*. (A) *In vitro* SUMOylation assay using IκBα WT or mutated on lysines 21 and 22 as substrates. (B) HEK293 cells were transfected with the indicated plasmids, pre-treated or not with MG132 and stimulated or not with TNFα. His_6_-ubiquitylated or SUMOylated proteins were purified using denaturing conditions and Ni^2+^ chromatography.

Since knockdown of Ubc9 does not provide information on which of the three SUMO molecules are involved in the regulation of IκBα, we investigated if in addition to SUMO-1 other SUMO molecules were able to modify IκBα. For this, we performed *in vitro* SUMOylation assays, which clearly indicate that IκBα WT, but not the IκBα mutant K21/22R, is modified by SUMO-1, SUMO-2 and SUMO-3 (Figure 2A). Similar results were obtained *ex vivo* after transfection of HEK293 cells with HA-IκBα-SV5 WT and histidine-tagged ubiquitin, SUMO-1, SUMO-2 or SUMO-3 (Figure 2B). In absence of stimulation, we observed a modification of IκBα by mono-modified forms of ubiquitin and all SUMO proteins. Akin to conjugation with SUMO-1 [21], these mono-modified forms of IκBα are likely to be independent of phosphorylation of the serine residues 32 and 36 since these forms occur in absence of TNFα (Figure 2B, left panel). After treatment with the proteasome inhibitor MG132 and stimulation with TNFα, we can observe an accumulation of high molecular weight bands, suggesting that polyubiquitylation and polySUMOylation of IκBα (mainly with SUMO-2) are significantly enhanced in this condition (Figure 2B right panel). Nevertheless, this approach does not exclude the possible integration of ubiquitin into SUMO chains and vice-versa, or simultaneous modification with both ubiquitin and SUMO on different lysines. Interestingly, the strongest band captured with His-ubiquitin, co-migrating with SUMO bands under unstimulated conditions, is the only one that appears to promote chain extension after TNFα stimulation, as indicated by its disappearance (marked with an asterisk in Figure 2B). Altogether these results suggest that all three SUMO modifiers have the capacity to modify exogenous IκBα to different extents, with SUMO-2 being the most evident under these experimental conditions (Figure 2B).

### Endogenous IκBα is Modified by SUMO-2/3*–*Ubiquitin Hybrid Chains

**Figure 3:**
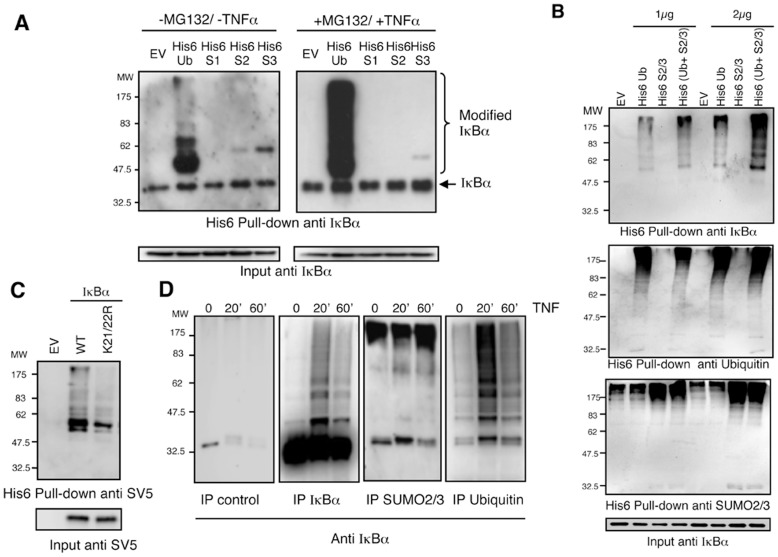
IκBα is modified by ubiquitin chains containing SUMO-2/3. (A) HEK293 cells were transfected with the indicated plasmids, pre-treated or not with MG132 and stimulated or not with TNFα. His_6_-ubiquitylated or SUMOylated proteins were purified using denaturing conditions and Ni^2+^ chromatography. (B) HEK293 cells were transfected with the indicated plasmids at two different concentrations 1 µg or 2 µg of each constructs. Empty vector (EV) was also used to compensate plasmid DNA to final concentration of 2 or 4 µg respectively. Cells were pre-treated with MG132 and stimulated with TNFα during the indicated times. His_6_-ubiquitylated or SUMOylated proteins were purified using denaturing conditions and Ni^2+^ chromatography. Captured material was analysed by western-blot with the indicated antibodies. (C) HEK293 cells were transfected with IκBα-SV5 WT or mutated on K21 and K22 in the presence of His_6_Ubiquitin, His_6_-SUMO2 and His_6_-SUMO3, pre-treated with MG132 and stimulated with TNFα. His_6_-modified proteins were purified using denaturing conditions and Ni^2+^ chromatography procedure (D) Time-course modification of IκBα after TNFα-stimulation analysed by immunoprecipitations with anti-IgG control, anti-ubiquitin, anti-IκBα and anti-SUMO-2/3 antibodies. Cells were treated with MG132, stimulated with TNFα and lysates were submitted to immunoprecipitation experiments as indicated. Precipitated material was analysed by western-blot with anti-IκBα antibody.

A similar approach was designed to capture endogenous IκBα in HEK293 cells transiently expressing histidinylated versions of ubiquitin, SUMO-1, SUMO-2 or SUMO-3 (Figures 3A, 3B). Under these conditions, we can observe, in the absence of TNFα stimulation, mono-modified forms of endogenous IκBα with SUMO-2 and SUMO-3 (Figure 3A). Nevertheless, chain extension on IκBα was only evident in cells transiently expressing His_6_-ubiquitin after 15 min stimulation with TNFα and in the presence of MG132 (Figure 3A). We interpreted these results as a handicap of His_6_-SUMO-2/3 to integrate into hybrid chains mainly composed of ubiquitin. We thus predicted that simultaneous co-expression of His_6_-tagged versions of both SUMO-2/3 and ubiquitin would lead to a cooperative effect during the purification, if hybrid chains exist on IκBα. Indeed, co-expression of His_6_-ubiquitin together with a mix of His_6_-SUMO-2/3 led to a clear increase of modified IκBα compared to expression of His_6_-ubiquitin or His6-SUMO-2/3 alone, indicating that both ubiquitin and SUMO-2/3 were simultaneously modifying this NF-κB inhibitor. The same results were obtained using two plasmid concentrations (Figure 3B). SUMOylated forms of IκBα captured with His_6_-SUMO-2/3 were only observed when the film was overexposed (data not shown). In agreement with our *in vitro* observations (Figure 2A), we found that K21 and K22 are the main residues involved in these modifications, since we observed a significant reduction of the capacity of the mutant IκBα K21/22R to capture SUMO-2/3-Ubiquitin heterologous chains (Figure 3C). The use of denaturing conditions in this protocol indicates that both modifiers are covalently linked to IκBα. These results were also confirmed by immunoprecipitations using anti-ubiquitin, anti-IκBα and anti-SUMO-2/3 antibodies followed by western-blot detection of IκBα (Figure 3D). Using this approach it can be observed that in the presence of the proteasome inhibitor MG132 and after 20 minutes of TNFα stimulation, IκBα accumulated as both ubiquitylated and SUMOylated forms. However, after 60 minutes of TNFα stimulation the levels of ubiquitylated IκBα dramatically decreased, while SUMOylated IκBα maintained a modest but consistent increase as compared to the unstimulated condition (Figure 3D).

**Figure 4:**
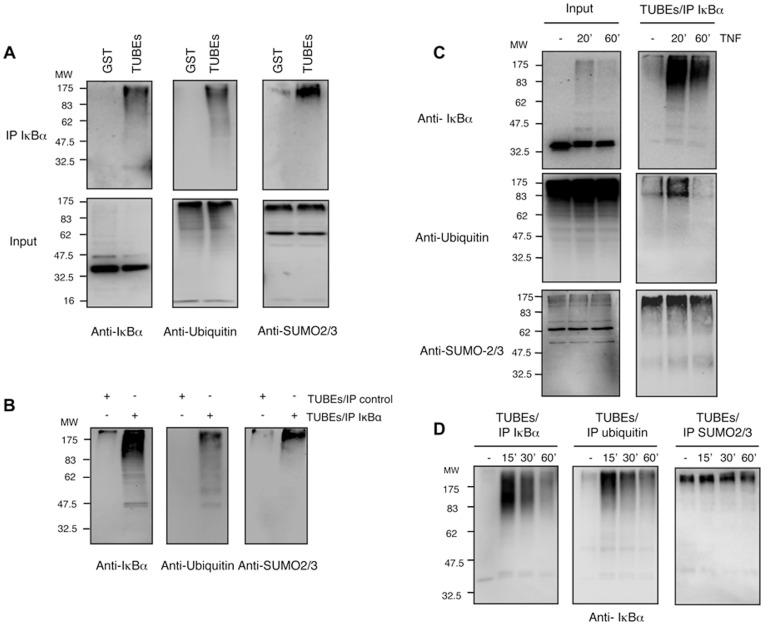
IκBα modified with hybrid SUMO-Ubiquitin chains is captured using Ubiquitin-traps. (A) HEK293 cells were pre-treated with MG132 and stimulated with TNFα for 20 min. Cells were lysed in a buffer containing TUBE-hHR23A or GST used as a control. GST-captured material was eluted and submitted to IκBα immunoprecipitation. (B) Cells were treated as in (A) and lysed in a buffer containing TUBE-hHR23A. Captured material was eluted and submitted to IκBα or control immunoprecipitations. (C) Cells were treated with MG132 and stimulated with TNFα for the indicated time. Cells were lysed in a buffer containing TUBE-hHR23A. Captured material was eluted and submitted to IκBα immunoprecipitation. (D) Cells were treated as in (C) and lysed in a buffer containing TUBE-hHR23A. Captured material was eluted and submitted to IκBα, ubiquitin or SUMO2/3 immunoprecipitations.

To further explore the role of heterologous SUMO2/3-ubiquitin chains in the regulation of IκBα stability after cell activation with TNFα, we used a tool recently developed by our group to capture endogenous ubiquitylated proteins. These ubiquitin-traps named TUBEs (Tandem Ubiquitin Binding Entities), specifically capture ubiquitin chains and do not directly bind to SUMO-1, SUMO-2, SUMO-3 or NEDD8 [27]. This technique respects the endogenous level of ubiquitin and ubiquitin-like molecules and therefore do not generate disequilibrium in other cellular functions when over-expression of these protein modifiers is used. Furthermore, this approach allows recovery of samples for further analysis when using a protocol coupled to an IκBα immunoprecipitation [28]. Our results show that the TUBEs-IP procedure efficiently purifies endogenous polyubiquitylated IκBα in a TNFα-mediated time-course response in HEK293 cells (Figure 4). Consistent with previously published observations [26], a peak of polyubiquitylated IκBα is detected after 20 minutes of TNFα stimulation (Figure 4C). However, polyubiquitylated IκBα is importantly reduced after 60 minutes of TNFα stimulation, even in the presence of proteasome inhibitors (Figure 4C). The observed signal is specific as GST or control antibody cannot capture polyubiquitylated IκBα (Figures 4A and 4B). To our surprise, under unstimulated conditions a basal level of polymodified IκBα was detected with the antibodies recognizing IκBα, ubiquitin and SUMO-2/3 antibodies (Figures 4C). Under these conditions, no significant signal was detected with anti SUMO-1 antibody (data not shown). The basal levels of IκBα ubiquitylation or SUMOylation are not artefacts of the TUBEs-mediated capture, as we do not observe this in non-activated rat tissues [29] (data not shown). Interestingly, antibodies recognizing SUMO-2/3 are able to specifically detect high molecular weight molecules progressively integrated within the chain architecture of polyubiquitylated IκBα (Figures 4C and 4D). It is important to note that while the increase of SUMOylation, as detected with anti-SUMO2/3 antibody appears to be modest, it does not decrease after 60 minutes of TNFα-stimulation, as is the case for the signal detected with the anti-ubiquitin antibody (Figures 4C). These findings also confirm the earlier results obtained by immunopreciptiation (Figure 3D) and suggest that IκBα captured after 60 minutes of TNFα-stimulation could correspond to ubiquitin chains enriched in SUMO-2/3. Thus taken together our data indicate that IκBα is modified by hybrid chains composed of SUMO-2/3 and ubiquitin.

### SUMO Molecules are Integrated within Ubiquitin Chains

**Figure 5:**
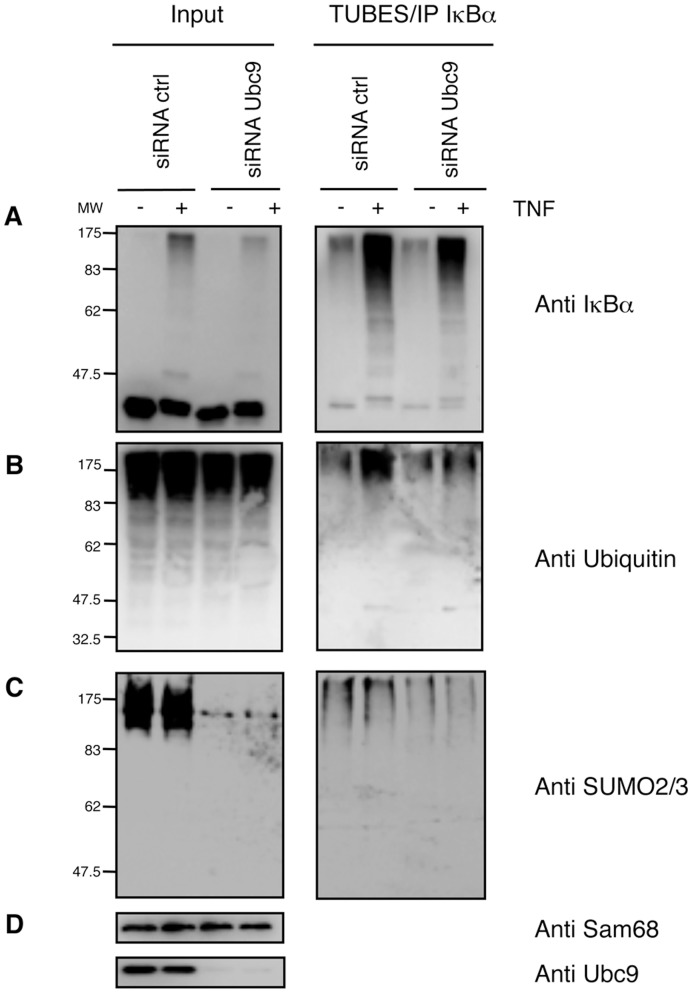
Integration of SUMO molecules into IκBα Ubiquitin chains. Seventy-two hours after transfection with control or Ubc9 siRNA (100 nM), HeLa cells were pre-treated with MG132, stimulated 20 min with TNFα and lysed in a buffer containing TUBE-hHR23A. TUBE-captured material was eluted and submitted to IκBα immunoprecipitation. Western blot detection with (A) anti-IκBα, (B) anti-ubiquitin, (C) anti-SUMO2/3 and (D) anti-sam68 and anti-Ubc9 antibodies.

In order to evaluate the contribution of all SUMO molecules in the architecture of ubiquitin chains modifying IκBα, we silenced Ubc9 and performed a TUBE-IκBα immunoprecipitation. This was done after 20 minutes of TNFα-stimulation thus coinciding with the peak of ubiquitylated IκBα, allowing us to analyse the contribution of SUMO-2/3 in the formation of hybrid chains. This timing will also favour the TUBEs-mediated capture of ubiquitylated species of IκBα. It was first confirmed that, while Ubc9 silencing reduces the input of SUMOylated molecules, it does not significantly affect the input of ubiquitylated substrates (Figures 5B and 5C). However, knockdown of Ubc9 led to decreased amounts of modified IκBα recovered with the TUBE-IP method, as detected with anti-IκBα and anti-ubiquitin antibodies (Figures 5A and B). Under the same experimental conditions western blot detections with anti SUMO-2/3 antibody show reduced levels of hybrid SUMO-2-ubiquitin chains (Figure 5C) despite the fact that the peak of IκBα SUMOylation is at 60 minutes of TNFα-stimulation (Figure 3D and Figure 4). Proportionally, the silencing of βTrCP has a higher impact on the capture of both ubiquitylated and SUMO-2-ubiquitin modified IκBα (data not shown), indicating that ubiquitin is a major component of the hybrid chains and confirming the heterologous nature of these chains.

### Ubiquitin and SUMO-2 Promote Efficient Chain Extension on IκBα

**Figure 6:**
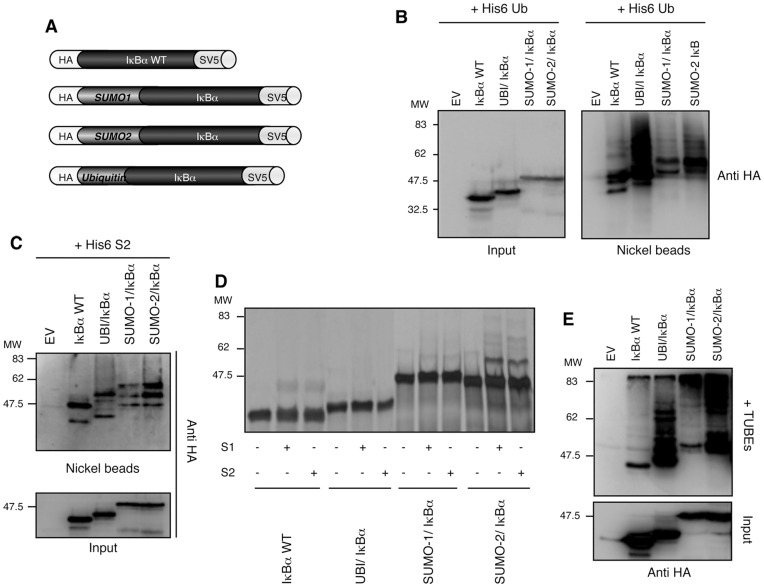
SUMO-2 and Ubiquitin promote efficient chain extension on IκBα. (A) Strategy used to make the different fusions proteins. (B) HEK293 cells were co-transfected with His6-ubiquitin and IκBα fusion-proteins as indicated. Cells were pre-treated with MG132 and stimulated 20 min with TNFα. His_6_-ubiquitylated proteins were purified using denaturing conditions and Ni^2+^ chromatography. EV: Empty Vector. (C) HEK293 cells were co-transfected with His6-SUMO-2 and IκBα fusions protein as indicated. Cells were pre-treated with MG132 and stimulated 20 min with TNFα as in A. His_6_-sumoylated proteins were purified using Ni^2+^ chromatography procedure. (D) *In vitro* SUMOylation assay using IκBα WT or fusion proteins as substrates. (E) HEK293 cells were transfected as indicated, pre-treated with MG132 and stimulated 20 min with TNFα. Cells were lysed in a buffer containing 3.5 µM of TUBE hHR23A. TUBE-captured material was eluted and submitted to IκBα immunoprecipitation. EV: Empty Vector.

To investigate if SUMO or ubiquitin molecules could promote chain extension on IκBα, chimeric proteins were obtained by fusing these modifiers to the N-terminus of IκBα. Since the conjugation site is very close to the IκBα N-terminus, we expect that the fusion protein will behave similarly to the endogenous SUMOylated protein. This strategy has been successfully used by several groups exploring the stability, localization and activity of diverse cellular factors [22], [30], [31]. Due to the high homology between SUMO-2 and SUMO-3 (86% identity) as well as the capacity of SUMO-2 to favour chain extension (Figure 2), we decided to develop a chimeric protein where IκBα was fused to SUMO-2 (Figure 6A). To make fusion proteins, the double C-terminal glycines (GG) were replaced by double alanines (AA) to avoid cleavage by DUBs or SUSPs. HA-N-terminal and SV5-C-terminal tags were included to monitor the integrity of the fusion proteins when expressed *ex-vivo*. To analyse the contribution of the fused moiety in the chain composition, chain extension and half-life of IκBα, fusions were made without the two lysines 21 and 22 necessary for IκBα ubiquitylation and SUMOylation. However, the presence of these lysines does not significantly alter the results obtained (data not shown). To determine the capacity of each fusion protein to be further modified by ubiquitin or SUMO, the different IκBα fusions were co-expressed with a histidinylated version of ubiquitin (Figure 6B) and SUMO-2 (Figure 6C) in HEK293 cells. As shown in Figure 6B, TNFα drives an efficient ubiquitin chain extension of both ubiquitin-IκBα and SUMO-2-IκBα fusions, far superior to the one observed with IκBαWT or SUMO-1-IκBα fusion. Similar observations were obtained when capturing SUMO-2 modified IκBα-fusions (Figure 6C). Whereas IκBαWT and SUMO-1-IκBα appear to be mainly mono-modified with SUMO-2, ubiquitin-IκBα and SUMO-2-IκBα are also polySUMOylated after 1hr pre-treatment with MG132 and TNFα stimulation (Figure 6C). However, a significant difference in the levels of ubiquitin-IκBα and SUMO-2-IκBα captured by His6-ubiquitin and His6-SUMO-2 indicate again that ubiquitin is a major component of the ubiquitin-SUMO-2 hybrid chains (compare Figures 6B and 6C).

The capacity of the different IκBα-fusion proteins to be modified with SUMO-1 and SUMO-2 was also analysed *in vitro* using SUMOylation assays. Although these assays lack any potential SUMO E3 for IκBα, resulting in a less efficient modification than *ex vivo* approaches, some differences can be observed. Only SUMO-2-IκBα was efficiently modified by SUMO-1 and SUMO-2 (Figure 6D). While a modest modification was observed for IκBαWT and SUMO-1-IκBα, no modification was detected on the ubiquitin-IκBα fusion. As IκBα lysines 21 and 22 are absent from all fusion proteins, the observed polySUMOylation of SUMO-2-IκBα might occur on the SUMO consensus site of SUMO-2 moiety [12]. The fact that the SUMO consensus is absent from SUMO-1 [32], suggests that when using overloaded *in vitro* conditions, additional lysine residues on SUMO-1 could be involved in this SUMOylation process [33]. To be able to capture ubiquitin-SUMO-2 hybrid chains from the distinct IκBα-fusions, we performed a TUBEs capture experiment. Results were similar to those obtained with the nickel beads approach using His6-ubiquitin or His6-SUMO-2, excepting that in the presence of TUBEs, very high molecular weight forms of SUMO-1-IκBα and SUMO-2-IκBα fusions were captured (Figure 6E). Under these experimental conditions, the bulk of ubiquitylated forms of IκBα appear to be shorter with ubiquitin-IκBα than the one obtained with the SUMO-IκBα fusions (Figure 6E). Thus, single ubiquitin and SUMO-2 moieties consistently promote further modification of IκBα with both ubiquitin and SUMO-2.

### SUMO2/3-Ubiquitin Heterologous Chains Drive an Efficient 26S Proteasomal Degradation of IκBα

**Figure 7:**
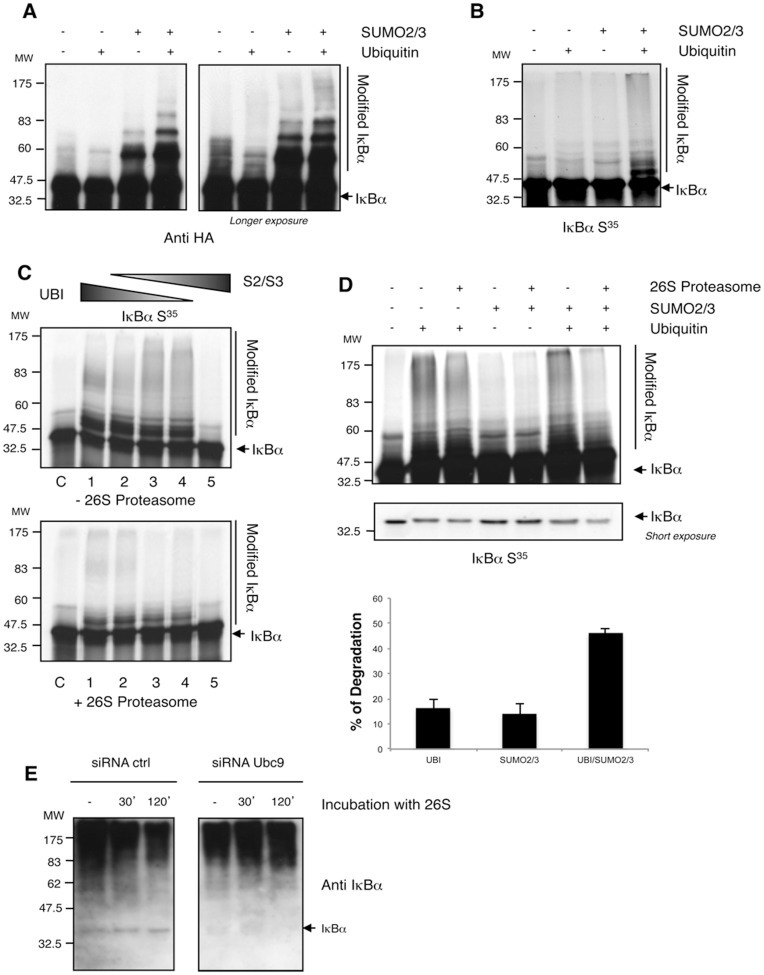
SUMO-2/3-Ubiquitin chains drive an efficient IκBα degradation by the 26S proteasome. (A) (B) *In vitro* ubiquitylation, SUMOylation or mixed assays using IκBα WT (A) or S^35^ IκBα WT (B) as substrates. Suboptimal conditions of conjugation were used in this assay (see materials and methods). (A) Western blot detection with the indicated antibodies. (B) Detection of radio-labelled material. (C) *In vitro* ubiquitylation, SUMOylation or mixed assays using S^35^ IκBα WT as substrate in the presence (+) or absence (-) of 26S proteasome. Saturating conditions of conjugation were used in this assay (see materials and methods). Different Ubiquitin: SUMO-2/SUMO-3 molar ratios were tested as follows: lane 1 = 4∶0/0, lane 2 = 3∶0.5/0.5, lane 3 = 2∶1/1, lane 4 = 1∶1.5/1.5, lane 5∶0:2/2. Detection of radio labelled material. (D) *In vitro* ubiquitylation, SUMOylation or mixed assays using S^35^ IκBα WT as substrate in the presence (+) or absence (−) of 26S proteasome. Replicated reactions using saturating conditions and the following ubiquitin: SUMO-2/SUMO-3 ratios: 4∶0/0 for lanes 1 and 2, 0∶2/2 for lanes 3 and 4 and 2∶1/1 for lanes 5 and 6. Phosphorimager quantification of modified forms of S^35^ IκBα WT in the presence or absence of 26S (n = 5). Standard deviation is indicated in the histograms. (E). Seventy-two hours after transfection with control or Ubc9 siRNA (100 nM), HeLa cells were pre-treated with MG132, stimulated with TNFα and lysed in a buffer containing TUBE-hHR23A. TUBE-captured material was submitted to IκBα immunoprecipitation. After IκBα-IP, extracts were eluted with glycine 200 mM pH2.5, equilibrated at pH 7.5 and submitted to an *in vitro* proteasome-mediated degradation assay at the indicated times.

As SUMO-2/3 appears to play a role in the formation of ubiquitin chains on IκBα, we decided to set up *in vitro* conjugation assays using all recombinant components and *in vitro* translated IκBα. In these assays, cell extracts from TNFα-stimulated HEK293 cells were used as source of E3s (see materials and methods). Using suboptimal conditions of conjugation, we observed by western blot (Figure 7A) or S^35^labelled IκBα (Figure 7B) that the simultaneous conjugation with ubiquitin and SUMO-2/3 allowed a more efficient hybrid chains formation on IκBα. However, the use of saturating conditions of conjugation does not always allow evaluation of cooperative effects of SUMO-2/3 on ubiquitin chain extension (Figure 7C and 7D). To investigate the role of ubiquitin-SUMO hybrid chains in proteasomal degradation of IκBα, *in vitro* modified material was submitted to degradation by the 26S proteasome. Different molar ratios of Ubiquitin: SUMO were tested to identify the optimal condition for IκBα modification and proteasomal degradation *in vitro*. We found that the ratios 2∶1/1 or 1∶1.5/1.5 of Ubiquitin: SUMO-2/SUMO-3 were the most efficiently modified (Figure 7C lanes 3 and 4). However, the ratio 2∶1/1 (lane 3) showed the best 26S-mediated proteasomal degradation of the modified IκBα (Figure 7C, bottom panel). Using these settings, we performed similar reactions with the same molar concentrations of Ubiquitin, SUMO-2/SUMO-3 or the combination of both. In this assay the abundant recombinant material (E1, E2 and modifiers) aims to out-compete but does not exclude the integration of SUMO or ubiquitin present in the transcription/translation reaction. While degradation of ubiquitin or SUMO-2/3 modified IκBα appears to be modest under these conditions (with only around 15% of the modified material been degraded), degradation of ubiquitin-SUMO hybrid chains is significantly more efficient and goes up to 46% (Figure 7D upper and bottom panels). These observations are also reflected in the amount of unmodified IκBα in a lower exposed film (Figure 7D middle panel). To confirm the contribution of SUMO-2/3 in the formation of ubiquitin chains that are driven to proteasomal degradation, SUMO-2/3-ubiquitin chains were purified using a TUBEs-IP IκBα protocol from control and siRNA Ubc9 cells. Immunopurified material was exposed to an *in vitro* degradation assay in the presence of purified 26S proteasome. Our results indicate that hybrid chains on IκBα promote a more efficient degradation of this protein after incubation with purified 26S proteasome as compared to the siRNA Ubc9 conditions (Figure 7E). These results also indicate that direct effects of SUMO-2/3 deficient ubiquitin chains on IκBα degradation can be evaluated using this method. Furthermore, under siRNA Ubc9 condition, we do not observe an *in vitro* deconjugation of IκBα, suggesting that de-modifying enzymes associated with the proteasome, are likely more efficient when SUMO molecules are integrated within the ubiquitin chains (Figure 7E). Cumulatively, these results are compatible with the proposed role of SUMO-2/3 molecules in the formation of ubiquitin chains and suggest that IκBα modification with SUMO-2/3 contribute to its optimal ubiquitin-dependent degradation by the proteasome in a similar manner as reported for PML [11], [12].

## Discussion

**Figure 8:**
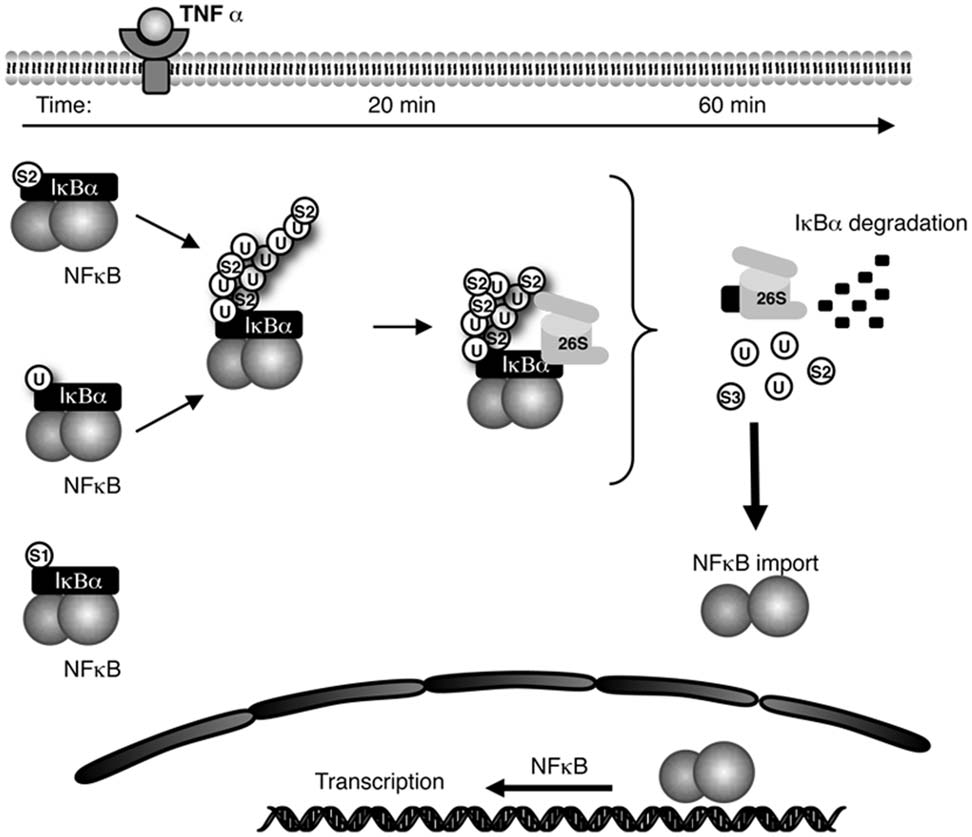
Integrated view of the time-dependent contribution of SUMO-2/3 in the formation of ubiquitin chains controlling the proteasomal degradation of IκBα and optimising NF-κB activity.

Considerable evidence underscores the significance of SUMOylation in the regulation of the transcription factor NF-κB [21], [22], [24]. Here, we demonstrate the importance of SUMO-ubiquitin hybrid chains in the TNFα-induced degradation of endogenous IκBα and activation of the NF-κB transcription factor (Figure 8). Multiple *in vitro* and *ex vivo* approaches such as the use of TUBEs and TUBEs/IP, Ubc9 silencing, Ni^2+^: NTA chromatography and reconstituted *in vitro* systems support these conclusions. We have shown that silencing of Ubc9 leads to loss of phosphorylation of IκBα, attenuation of SUMO-2-Ubiquitin heterologous chains on IκBα, decreased proteasomal degradation of IκBα and a delay in NF-κB activation. Nevertheless, the role of SUMO in NF-κB signalling is difficult to integrate into a simple model, mainly due to its capacity to act at different levels of this pathway [21], [22], [24]. Although the stability of other known SUMO targets such as p100 or IKKγ/NEMO were not affected by the Ubc9 silencing, we cannot exclude that the function of these SUMO targets is altered or that other unidentified SUMO substrates regulate the stability of IκBα and NF-κB-dependent transcription. For instance, upon genotoxic stress the SUMO ligase PIASy induces the modification of IKK with SUMO-1 but not SUMO-2/3, resulting in an increased NF-κB activity [34]. One could speculate that the inhibition of Ubc9 could have affected IKK activity by reducing its SUMOylation, repressing IκBα degradation and consequently acting on NF-κB activity. However, SUMOylation has also been associated to repression of RelA/p65 nuclear translocation [35], making difficult to conclude that Ubc9 silencing will always promote repression of NF-κB activity. An important piece of evidence is that TUBE captures endogenously modified IκBα, mainly composed by ubiquitylated and SUMO-2/3 conjugated forms of IκBα. TUBEs-captured IκBα behave as previously reported with a peak of ubiquitylated IκBα after 20 minutes of TNFα stimulation followed by a reduction at 60 minutes, even in the presence of the proteasome inhibitor MG132 [26]. Remarkably after 60 minutes of TNFα stimulation and proteasome inhibitor treatment, the proportion of IκBα modified by SUMO-2/3 is not significantly reduced. These results suggest that the fraction of IκBα modified by hybrid SUMO-Ubiquitin chains is resistant to deubiquitylating enzymes (DUBs). Consistent with these results IκBα mainly modified with SUMO-2/3 chains is a poor 26S proteasome substrate.

Although our results clearly demonstrate that SUMO-2/3 favours the covalent modification of IκBα with ubiquitin, the order of chain formation needs to be further investigated. Due to the inefficiency of ubiquitin to prime SUMO-2 chain extension *in vitro*, one can speculate that SUMO-2 could be attached first to somehow predispose the modified IκBα to ubiquitylation, playing a potential role of ubiquitin-chain extender. The SUMO-2-priming ubiquitylation model is also consistent with the fact that a monoubiquitylation of IκBα induces time-dependent resistance to proteolysis [29]. However, the fact that SUMO-2/3 better integrates ubiquitin chains than ubiquitin integrates SUMO-2/3 chains, suggest that both protein modifiers have the potential to prime and extend chains. Conclusions on the order of integration into the ubiquitin chains have to be taken with caution as information, recently published, indicates that over-expression of ubiquitin-like proteins or stresses such as inhibition of the proteasome activity can force the ubiquitin conjugating enzymes to incorporate ubiquitin like proteins into ubiquitin chains [36], [37], [38]. The existence of one main lysine receptors for SUMOylation (K21) indicates that if SUMO-2/3 is attached first to IκBα, ubiquitin should be attached on it to generate chain extension. If ubiquitin is attached first to IκBα, two ubiquitin-SUMO mixed chains are most likely to be formed on K21/K22. However, one other hypothetical possibility is that there is a mixed population of independently SUMOylated (on K21) and Ubiquitylated (on K22) IκBα as it is illustrated in Figure 8. Mass spectrometry data should put some light on the architecture of Ubiquitin-SUMO-2/3 chains modifying IκBα. A major technical bottleneck is the identification of SUMO peptide signatures attached to endogenous proteins. This is reflected by the fact that only a bit more than a hundred SUMOylation sites have been identified [39], [40].

All together our data suggest that SUMO2/3 can be incorporated into ubiquitin-chains to regulate IκBα proteasomal degradation. Beyond the observation that the optimal proteasome degradation of IκBα is mediated by hybrid SUMO-ubiquitin chains conditioning the activity of NF-κB, the integration of SUMO-2/3 into the hybrid SUMO-ubiquitin chains could simultaneously favours ubiquitin chain extension and recycling of IκBα (Figure 7). In apparent contradiction, chains enriched with SUMO-2/3 also appear to promote proofreading/recycling of IκBα. Therefore our data propose the existence of a mechanism regulating the balance between SUMO-2/3 and ubiquitin into the same chain, offering the possibility to regulate proteasome-mediated proteolysis and resistance to the action of DUBs. Although some aspects of this speculative working model (Figure 8) require further investigation, it suggests the presence of a dynamic way to regulate protein degradation, proofreading, recycling of molecules and recovery of the cytoplasmic reservoir of NF-κB/IκBα, typical of this highly dynamic system.

Under hypoxic conditions SUMOylation of IκBα is also regulated [41], [42] indicating that different types of stimulation might influence the level of IκBα SUMOylation. Other SUMO substrates are regulated under different stress conditions such as heat shock, proteasome inhibition or chemotherapeutic drugs [39], [43], [44]. The best-known protein regulated by SUMO-2/3 is the promyelocytic leukemia protein PML whose degradation is induced by Arsenic Trioxide (ATO) [11], [12]. As occurs for IκBα, the formation of chains containing Ubiquitin and SUMO-2/3 drive PML to proteasomal degradation [11], [12]. The mechanism driving the formation of these hybrid chains on PML is well characterized and involves the action of the SUMO-dependent ubiquitin ligase RNF4 [11], [12]. However, in the case of IκBα it is not clear if the mechanism is similar since SUMO-2/3 does not favour the integration of ubiquitin moieties after TNFα treatment as ATO does it for PML (Data not shown). Furthermore, RNF4 SIM domains do not capture SUMOylated IκBα after proteasome inhibition with MG132 and TNFα-stimulation (data not shown). Whether the mechanism of IκBα degradation involves the participation of a different SUMO-dependent ubiquitin-protein ligase or ubiquitin-dependent SUMO-ligase will have to be investigated.

Thus, the proteolytic mechanisms involving hybrid SUMO-Ubiquitin chains is not restricted to PML. The evidence presented here underlines the contribution of SUMO-2/3 in the control of IκBα degradation. This time-dependent formation of high molecular weight ubiquitin-SUMO-2/3 chains likely optimises IκBα proteasomal degradation and controls NF-κB activity (Figure 8). The mechanism of SUMO-ubiquitin hybrid chains could be more general and might affect more protein targets than initially suspected [45].

## Materials and Methods

### Cell Culture and Cell Based Assays

HEK293 and HeLa (ATCC) cells were grown in DMEM (Gibco) supplemented with 10% FBS and antibiotics. Cells transfections were done using lipofectamine following manufacturer instructions (Invitrogen). Depletion of endogenous Ubc9 expression was achieved by RNA interference. Small interfering RNAs (siRNAs) used for human Ubc9 knock down were already validated [23], [46]. HeLa cells were transfected with either scrambled, or Ubc9 siRNAs (100 nM per well) using lipofectamine 2000 (Invitrogen) according to the manufacturer’s instructions. After 72-h culture, cells were treated as indicated. For the luciferase experiment, HeLa cells were transfected with a NF-κB-luciferase reporter plasmid (3-EnhConA) and a pSV-β-galactosidase reporter 24h before the luciferase and β-galactosidase measurements.

### Immunodetections

Western blot detections were performed with the following primary antibodies: mouse monoclonals HA (Covance); Ubiquitin (P4D1, Santa Cruz; FK2, ENZO); SUMO-1 [13]; IκBα (Cell Signalling Technology); GAPDH (Sigma) antibodies and rabbit polyclonals IκBα (Santa Cruz Biotechnology); IKKγ/NEMO (Cell Signalling Technology); p100/p52 (Cell Signalling Technology); SUMO2/3 (kindly provided by Dr Alfred Vertegaal and Paul Fraser); Sam68 (Santa Cruz Biotechnology) antibodies. Ubc9 antibody was used as previously described [12]. Immunoprecipitation experiments were performed using for IκBα (Cell signalling) 4µg of antibody/point, for SUMO2/3 (kindly provided by Dr Paul Fraser) 7µg/point and for ubiquitin (FK2, ENZO) 3µg/point. Immunoprecipitation experiments were performed using Protein-G cross-linked with the anti-IgG control, anti-IκBα, anti- ubiquitin or anti-SUMO2/3 antibodies. In all cases, cells were lysed for 15 minutes on ice in 50 mM sodium fluoride, 5 mM tetra-sodium pyrophosphate, 10 mM beta-glyceropyrophosphate, 1% Igepal CA-630, 2 mM EDTA, 20 mM Na_2_HPO_4_, 20 mM NaH_2_PO_4_, 1 mM Pefablock, 1.2 mg/ml Complete protease inhibitor cocktail (Roche).

### Cloning

Ubiquitin, SUMO-1, SUMO-2 (accession numbers CAA44911, NM-003352 and NM-006937 respectively) were used to generate IκBα fusions and cloned into BamHI/Not1 restriction sites of a modified pcDNA3 vector containing a N-terminal HA tag and C-terminal SV5 tag. The C-terminal glycine residues (GG) of SUMO-1, SUMO-2 or ubiquitin were changed to alanine (AA), and lysine 21 and 22 of IκBα were mutated to alanine to avoid respectively the action of DUBs and additional attachment of moieties at the N-terminus of IκBα using the following oligonucleotides: for ubiquitin fusion proteins 5′-ctc cgt ctt aga gct gcg gag cgg cta ctg gac gac-3′ and 5′-gtc gtc cag tag ccg ctc cgc agc tct aag acg gag-3′, for SUMO1 5′-cag gaa caa acg gcg gct gag cgg cta ctg gac gac-3′, and 5′-gtc gtc cag tag ccg ctc agc cgc cgt ttg ttc ctg-3′ and for SUMO-2 5′-caa cag gag acg gca gct gag cgg cta ctg gac gac-3′and 5′-gtc gtc cag tag ccg ctc agc tgc cgt ctg ctg ttg-3′. As a control, a construct of IκBα WT, containing an N-terminal HA tag and C-terminal SV5 tag, was used (Figure 6A). All constructs have been verified by DNA sequencing. His-6-Ubiquitin, His-6-SUMO-1, His-6-SUMO-2 and His-6-SUMO-3 have been previously reported [47].

### Purification of SUMO and Ubiquitin Chains

His_6_-ubiquitylated or SUMOylated proteins were purified using denaturing conditions and Ni^2+^ chromatography as previously described [48]. The use of low-density nickel beads (QLNI-100, ABT) reduced the capture of sticky unmodified IκBα (Figure 3B) compared to high-density beads (QLNI-25, ABT) (Figures 2B and 3A). However, it also reduces the purification of poorly expressed IκBα monoSUMOylated forms (Figure 3A). To capture ubiquitin chains using TUBEs, the lysis buffer was supplemented either with 3.5 µM of TUBEs hHR23A or GST as previously described [27], [28]. Lysates were clarified by cold centrifugation, and added to glutathione agarose beads (Sigma). Glutathione beads were eluted and bound material was submitted to western blot analysis or to IκBα, ubiquitin or SUMO2/3 immunoprecipitations.

### *In vitro* 26S-mediated Degradation Assay of TUBE-captured IκBα

Polyubiquitylated proteins were captured using TUBEs as described [28]. Samples were briefly eluted from TUBEs using a glycine buffer pH 4 and equilibrated with Tris pH 7.5 to a final concentration of 100 mM. Eluted material was immunoprecipitated using a specific IκBα antibody. Ubiquitylated IκBα was eluted from specific antibodies with glycine pH 2.5, neutralized as indicated previously, before being submitted to an *in vitro* degradation assay as reported [27] using 2 µg of 26S proteasome (ENZO) for 30 and 120 minutes at 37°C.

### *In vitro* SUMOylation, Ubiquitylation and Hybrid Ubiquitylation*-*SUMOylation Assays

For the SUMOylation assays, *in vitro* transcribed/translated IκBα (^35^S-Met-labelled or not when indicated) were incubated in a buffer containing an ATP regenerating system [(50 mM Tris pH 7.5, 10 mM MgCl2, 2 mM ATP, 10 mM creatine phosphate (Sigma), 3.5 U/ml of creatine kinase (Sigma), and 0.6 U/ml of inorganic pyrophosphatase (Sigma)], SUMO-1, 2 or 3 (1 µg), Ubc9 (0.325 µg) and purified SAE1/2 (0.8 µg, ENZO Life Sciences). When suboptimal conjugation conditions were used, the amount of SUMO-2, SUMO-3, SAE1/2, and Ubc9 were reduced to half (Figure 7A and 7B).

For the ubiquitylation assays, *in vitro* transcribed/translated IκBα (^35^S-Met-labelled or not when indicated) were incubated in a 15 µl reaction including an ATP regenerating system [25 mM Tris pH 7.5, 5 mM MgCl2, 2 mM ATP, 10 mM creatine phosphate (Sigma), 5 mM NaCl_2_, 3.5 U/ml of creatine kinase (Sigma) and 0.6 U/ml of inorganic pyrophosphatase (Sigma)], 1 µg ubiquitin (Sigma), 10 ng human E1 (ENZO Life Sciences), 500 ng UbcH5b (ENZO Life Sciences). When suboptimal conjugation conditions were used, the amount of Ubiquitin, Ubiquitin E1, and UbcH5b were reduced to half (Figure 7A and 7B). Suboptimal conditions were used to observe SUMO-Ubiquitin cooperative effects. When indicated, different ratios of Ubiquitin/SUMO-2/SUMO-3 were tested.

For the hybrid ubiquitylation*-*SUMOylation assays, *in vitro* transcribed/translated IκBα (^35^S-Met-labelled or not when indicated) were incubated in a buffer reaction including an ATP regenerating system (see *In vitro ubiquitylation assay*) supplemented with 2 µg ubiquitin (Sigma), 10 ng human E1 (ENZO Life Sciences), 500 ng UbcH5b (ENZO Life Sciences), SUMO-2 and SUMO-3 (1 µg each), Ubc9 (0.325µg) and purified SAE1/2 (0.8µg, ENZO Life Sciences). When suboptimal conjugation conditions were used, the amount of Ubiquitin, SUMO-2, SUMO-3, Ubiquitin E1, UbcH5b, SAE1/2, and Ubc9 were reduced to half (Figure 7A and 7B). Suboptimal conditions were used to observe SUMO-Ubiquitin cooperative effects. When indicated, different ratios of Ubiquitin/SUMO-2/SUMO-3 were used.

In some cases (Figure 7), reactions were supplemented with 1µg of cytoplasmic extracts from HEK293 cells, stimulated during 20 min with 10 ng/ml of TNFα, as a source of E3 enzymes. Reactions were incubated at 30°C for 2 hours and stopped by addition of SDS sample buffer. Reaction products were resolved by SDS-PAGE (12%) and dried gels analysed by autoradiography.

### *In vitro* 26S Proteasome-mediated Degradation Assay

^35^S methionine-labelled *in vitro* transcribed/translated IκBα were submitted to *in vitro* ubiquitylation, SUMOylation or hybrid chains modification assays in the presence or not of 3 µg of purified human 26S proteasomes [49] [27]. Reactions were incubated at 30°C for 2 hours and stopped by addition of SDS sample buffer. Reaction products were resolved by SDS-PAGE and dried gels analysed by phosphorimaging.
